# On-chip mid-infrared silicon-on-insulator waveguide methane sensor using two measurement schemes at 3.291 μm

**DOI:** 10.3389/fchem.2022.953684

**Published:** 2022-08-23

**Authors:** Huan Zhao, Chuantao Zheng, Mingquan Pi, Lei Liang, Fang Song, Yu Zhang, Yiding Wang, Frank K. Tittel

**Affiliations:** ^1^ State Key Laboratory of Integrated Optoelectronics, College of Electronic Science and Engineering, Jilin University, Changchun, China; ^2^ State Key Laboratory of Luminescence and Applications, Changchun Institute of Optics Fine Mechanics and Physics, Chinese Academy of Sciences, Changchun, China; ^3^ Department of Electrical and Computer Engineering, Rice University, Houston, TX, United States

**Keywords:** methane sensing, SOI waveguide sensor, direct absorption spectroscopy, wavelength modulation spectroscopy, kalman filter

## Abstract

Portable or even on-chip detection of methane (CH_4_) is significant for environmental protection and production safety. However, optical sensing systems are usually based on discrete optical elements, which makes them unsuitable for the occasions with high portability requirement. In this work, we report on-chip silicon-on-insulator (SOI) waveguide CH_4_ sensors at 3.291 μm based on two measurement schemes including direct absorption spectroscopy (DAS) and wavelength modulation spectroscopy (WMS). In order to suppress noise, Kalman filter was adopted in signal processing. By optimizing the waveguide cross-section structure, an etch depth of 220 nm was selected with an experimentally high power confinement factor (PCF) of 23% and a low loss of only 0.71 dB/cm. A limit of detection (LoD) of 155 parts-per-million (ppm) by DAS and 78 ppm by WMS at an averaging time of 0.2 s were obtained for a 2 cm-long waveguide sensor. Compared to the chalcogenide (ChG) waveguide CH_4_ sensors at the same wavelength, the reported sensor reveals the minimum waveguide loss and the lowest LoD. Therefore the SOI waveguide sensor has the potential of on-chip gas sensing in the mid-infrared (MIR) waveband.

## 1 Introduction

Methane (CH_4_) is regarded as the second most important greenhouse gas after carbon dioxide (CO_2_), responsible for about 20% of the global warming induced by greenhouse gases emissions (Shindell et al., 2009; Howarth et al., 2011; Kirschke et al., 2013). Due to the exploitation of fossil fuels, such as coal, oil and natural gas, atmospheric CH_4_ concentration levels are rising in recent years (Zheng et al., 2017). In addition, CH_4_ is explosive when mixed with 5–15% volume of air, which may become an industrial safety hazard (Leis et al., 2014). Hence, it is necessary to monitor CH_4_ concentration level effectively for environmental protection and production safety (Berman et al., 2012; Sur et al., 2015; Ye et al., 2016). Compared with the sensing system based on discrete optical elements, on-chip optical waveguide sensors are small in device footprint, which have the potential to integrate light source and detector on a single chip (Dong et al., 2018; Ranacher et al., 2018; Zheng et al., 2018; Pi et al., 2019).

Several approaches based on optical waveguide have been proposed to detect analyte concentration, which can be broadly grouped into two categories: refractive index sensing (El Shamy et al., 2019; Soref et al., 2019) and optical absorption sensing (Ranacher et al., 2016; Qiao et al., 2019). Refractive index sensing is based on measuring the change in the refractive index of the analyte, which would result in the change in frequency or phase of the output light (Zhang et al., 2019). Compared to refractive index sensing, waveguide sensors based on optical absorption spectroscopy are selective since each analyte has unique absorption spectrum. By detecting the light attenuation when the light with specific wavelength passes through the analyte, the concentration of the analyte can be determined (Liu et al., 2016; Khonina et al., 2020). In order to reduce the limit of detection (LoD), according to Lambert-Beer Law, it is necessary to increase the optical path length, that is, the length of interaction between light and analyte. For optical waveguide sensors, analyte acts as cladding of the waveguide and interacts with the evanescent field of the waveguide (Butt et al., 2017; Tombez et al., 2017; Kita et al., 2018; Yoo et al., 2020). Power confinement factor (PCF) indicates the ratio of light power in the analyte cladding layer and a higher PCF means that the light can interact with the analyte sufficiently (Gutierrez-Arroyo et al., 2017). Considering the relatively large transmission loss, waveguide cannot be made very long, so the PCF should be enhanced to increase the equivalent optical path length.

CH_4_ has stronger absorption in the mid-infrared around 3.3 and 7.6 μm as when compared to the absorption in the near-infrared. Chalcogenide (ChG) glasses are usually used in the MIR due to their low absorption loss (Rao et al., 2015; Zhang et al., 2018). In addition to chalcogenide glasses, silicon-on-insulator (SOI) is also a potential material platform for mid-infrared CH_4_ sensing, since it is transparent in the absorption waveband of CH_4_ (3.2–3.45 μm) (Hu et al., 2017; Zou et al., 2018). By optimizing the cross-section structure of SOI waveguide, a higher PCF can be obtained. In this paper, we proposed a high-performance SOI waveguide CH_4_ sensor based on two measurement schemes, i.e. direct absorption spectroscopy (DAS) and wavelength modulation spectroscopy (WMS). Combined with wavelength modulation spectroscopy (WMS), system noise can be reduced, leading to a decrease of LoD (Liu et al., 2019). At the same time, in order to further suppress the system noise, Kalman filter was adopted. The PCF was experimentally obtained with an accurate calculation method and related formulations as well as equations were derived. CH_4_ sensing performances were presented and discussed in detail based on the two schemes.

## 2 Design and fabrication of mid-infrared silicon-on-insulator waveguide

### 2.1 Waveguide sensor structure and fabrication

**FIGURE 1:**
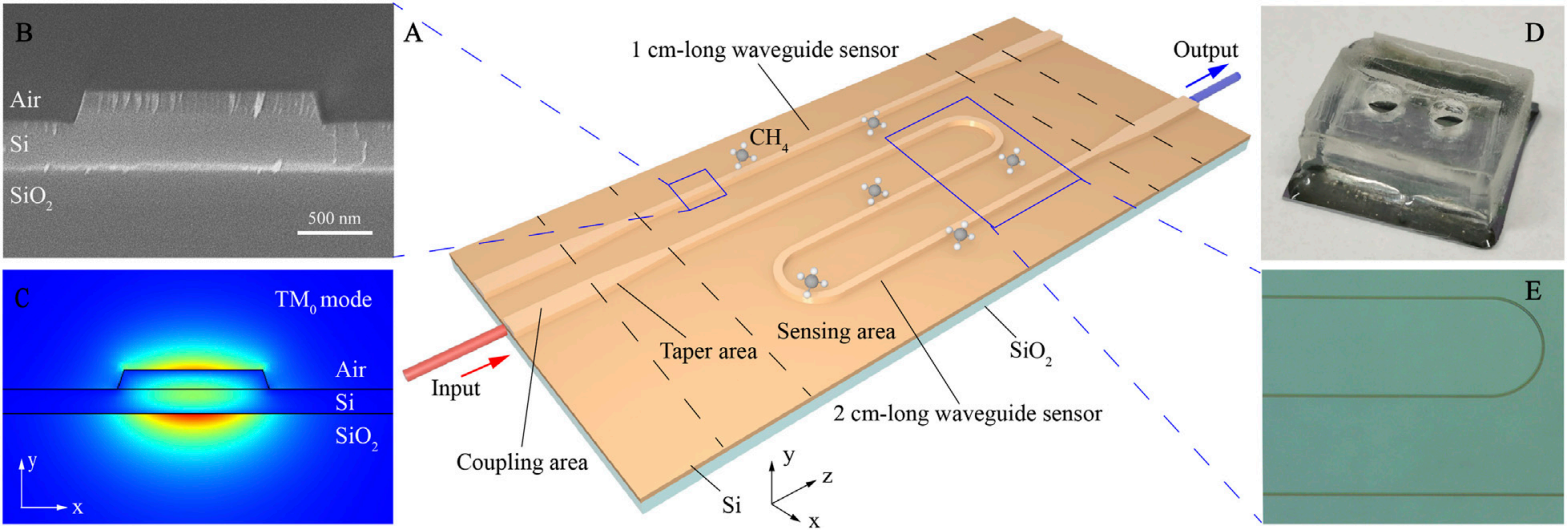
**(A)** Schematic of the SOI ridge optical waveguide sensor chip, which is composed of sensing area, taper area and ridge waveguide at both ends for coupling with optical fiber. There are two waveguide sensors on the chip: a straight waveguide sensor with a length of 1 cm and a bend waveguide sensor with a length of 2 cm. **(B)** SEM image of the cross section of a fabricated SOI waveguide. **(C)** The simulated fundamental TM mode of the SOI waveguide. **(D)** Photograph of the SOI waveguide with a gas cell. **(E)** Micrograph of the sensing area.

The structure of the SOI ridge optical waveguide sensor is shown in Figure 1A. The optical waveguide consists of a central sensing region and a coupling region at both ends of the chip. Since the core diameter of the optical fiber is 7.5 μm, in order to realize the coupling with the optical fiber and reduce mode mismatch, the ridge width of the waveguide at both ends is 15 μm. The length of the tapered transition waveguide is 300 μm. To reduce the footprint of the sensing area, we designed a 1 cm-long straight waveguide and a 2 cm-long bend waveguide, which can be tested on the same chip. The radius of the bend waveguide is 50 μm to reduce bending loss. Figure 1B shows the cross-sectional scanning electron microscope (SEM) image of the fabricated SOI ridge optical waveguide in the sensing area. The SOI waveguide was photolithographed with AZ701 photoresist. After the development, the silicon device layer on the top of the SOI chip was etched by inductively coupled plasmonic (ICP) with CF_4_ gas. The etching rate is 220 nm/min. The thickness of the Si layer is 500 nm and the etching depth of the waveguide is 220 nm. Due to the limitation of etching process, the waveguide sidewall is not completely steep, so the etching area is trapezoid, in which the upper bottom width is 1.5 μm and the lower bottom width is 1.65 μm.

The cross-sectional mode field distribution of the SOI ridge waveguide is simulated by COMSOL Multiphysics. The refractive index of Si and SiO_2_ at the wavelength of 3,291 nm are 3.4302 and 1.4119, respectively. The output mode field of the mid infrared laser is TM polarized. In order to test the polarization of the light before coupling into the waveguides, we placed a polarizer behind the fiber and tested the output power under different polarization states. Test results show that the output power under TM and TE polarization is 54.3 and 12.9 μW, respectively, and the total power without polarizer is 79.3 μW. It can be seen that the light output from the optical fiber is mainly TM polarized, so we mainly focus on TM mode distribution of the waveguide. Figure 1C shows the TM_0_ mode distribution of SOI waveguide section with an effective refractive index of 1.889. Figure 1D shows the photograph of the SOI waveguide with a gas cell bonded on it. Micrograph of the sensing area is shown in Figure 1E in detail.

### 2.2 Experimental setup and details for on-chip CH_4_ sensing

**FIGURE 2:**
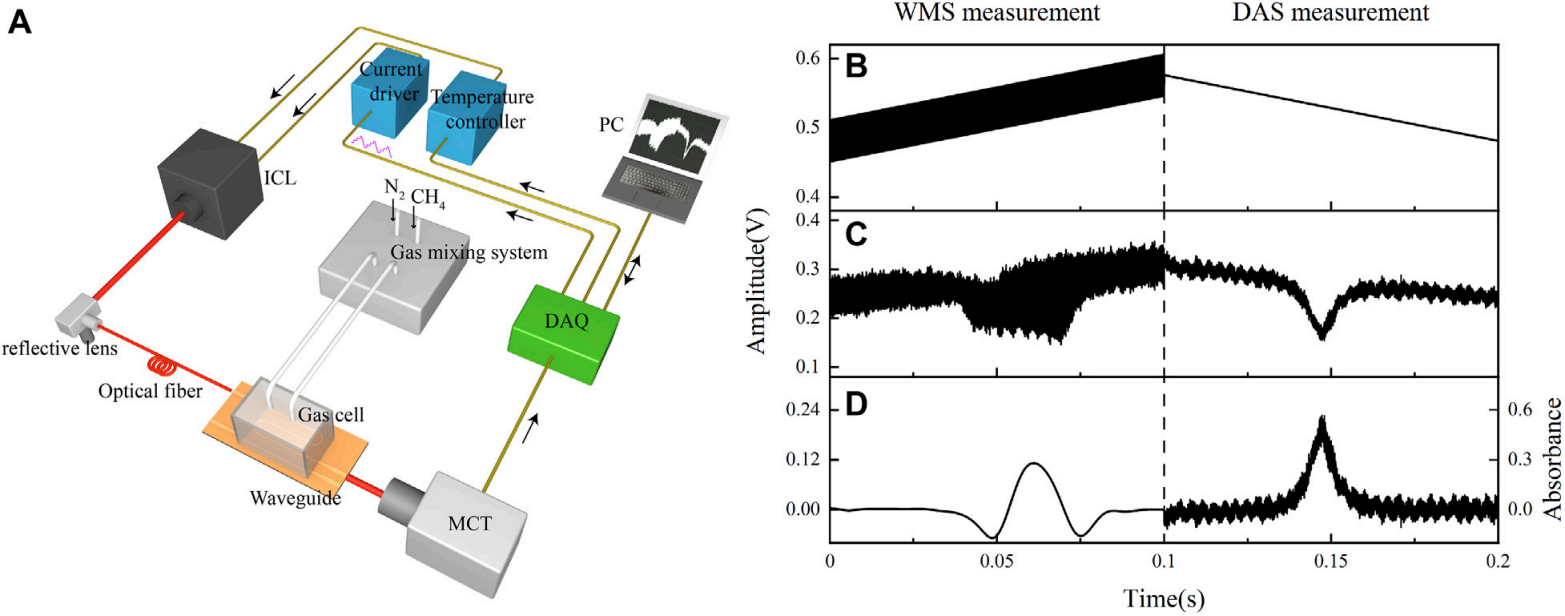
**(A)** Schematic of the SOI waveguide sensor system. **(B)** Driving signal of the ICL. For the rising edge, a ramp signal is superimposed with a high-frequency sine wave signal for WMS measurement. For the falling edge, only a ramp signal is used for DAS measurement. **(C)** The output sensing signal from the detector and **(D)** the extracted 2f signal and absorbance signal under a CH_4_ concentration level of 40% (4 × 10^5^ ppm).

The CH_4_ sensing system based on SOI optical waveguide is shown in Figure 2A. An ICL (Nanoplus) emitting at 3,291 nm was used as the light source, which can cover the absorption line of CH_4_ by changing its driving current. A polydimethylsiloxane (PDMS) gas cell was integrated on the SOI chip, and the gas inflow and outflow were carried out through the inlet and outlet above the cell. The width of the input and output coupling waveguides is 15 μm, and there is little evanescent field leaking above the waveguide, which can reduce the light absorption of the PDMS gas cell. The pressure in the gas cell was not controlled during experiment, and the measurement was conducted under ambient pressure of 760 Torr (Pi et al., 2021). Through a fluoride fiber, the light from the ICL was coupled into the SOI waveguide, and the light from the waveguide was directly incident into a mercury-cadmium-telluride (MCT) detector (VIGO System) through free-space, which can convert the optical signal to an electrical signal. Quasi-simultaneous DAS and WMS were used to detect CH_4_ concentration. Through the LabVIEW-controlled data acquisition (DAQ) card, the triangular wave signal for driving the laser was generated. The first half cycle of the signal is used for WMS, in which the rising ramp signal is superimposed with the high-frequency sine wave signal. The second half cycle of the signal is used for DAS and is a falling slope signal used for wavelength scanning. The LabVIEW based lock-in amplifier was used to extract the second harmonic (2f) signal from the first half cycle of the output signal from the detector. At the same time, a LabVIEW-based signal processing program was used to process the absorption signal of the second half cycle. In order to suppress the signal fluctuation caused by air flow on the surface of the waveguide, Kalman filter was added to the signal processing program. In the later gas detection experiments, the effect of the filter on improving the sensitivity of gas detection will be verified. Figures 2B–D show the flow chart of the signal processing using WMS and DAS. Figure 2B shows the driving signal of the ICL. The output sensing signal from the detector is shown in Figure 2C. Figure 2D shows the extracted 2f signal and absorbance signal under a CH_4_ concentration level of 40% (4 × 10^5^ ppm).

### 2.3 Sensing theory

The light of the evanescent field above the waveguide is absorbed by the target gas. The gas concentration can be obtained by monitoring the change of the output light intensity. In addition, the intrinsic optical absorption of waveguide materials needs to be considered. The basic sensing theory of the waveguide gas sensor obeys the Lambert-Beer Law (Schilt et al., 2003; Siebert and Muller, 2005; Charrier et al., 2012)

$$I_{\text{out}}=I_{\text{in}}\exp\left(-\Gamma \alpha_{{\text{CH}}_{4}}cL-\alpha_{\mathrm{int}}L\right)$$ (1)

where *I*_out_ and *I*_in_ are the output and input light intensity, *α*_CH4_ is the absorption coefficient of CH_4_, *α*_int_ is the intrinsic loss of the waveguide, *L* is the waveguide length of the sensing area, and *c* is the concentration of the gas analyte. Since *α*_CH4_ is the absorption coefficient per unit CH_4_ concentration and length, it needs to be multiplied by the actual CH_4_ concentration *c* and waveguide length *L*. In addition, on the waveguide transmission section, only the light of the evanescent field in the upper cladding is absorbed by the gas, so it needs to be multiplied by the power ratio of the evanescent field. *Γ* is the power confinement factor (PCF) in the upper cladding of the waveguide, which can be expressed as (Robinson et al., 2008; Vlk et al., 2021)

Γ=ng∬clε|E|2dxdyRe{ncl}∫∫−∞∞ε|E|2dxdy (2)

Here, *n*_cl_ is the refractive index of gas, *ε* is the permittivity, and *E* is the electric field, *n*_g_ is the group refractive index which captures the waveguide dispersion. For the designed SOI waveguide, only TM_0_ and TM_1_ modes can be transmitted. In the calculation of PCF, the dominant TM_0_ mode is considered under the incident way of the laser mode. *n*_g_ of the TM_0_ mode is 5.18, and the PCF was numerically calculated to be 34.3%.

### 2.4 Transmission loss of the silicon-on-insulator waveguide

The transmission loss of the SOI waveguide can be measured by ‘cut-back’ method, with the expression of

$$\alpha_{\mathrm{int}}=10\frac{\log_{10}\left(I_{\text{out}2}/I_{\text{out}1}\right)}{L_{1}-L_{2}}$$ (3)

where *L*_1_ (i.e., 1 cm) and *L*_2_ (i.e., 2 cm) are the lengths of the two different waveguides, *I*_out1_ and *I*_out2_ are the output light intensity from the corresponding waveguides. By measuring the ratio between the output power of the two different waveguides, the transmission loss of the waveguide can be obtained. Here we measured the output signal amplitude of the two waveguides to calculate the signal amplitude ratio, which are equal to the ratio of intensity. N_2_ was continuously injected into the gas cell, the modulation signal amplitude is set to 0, and the lengths of two waveguides are 1 and 2 cm, respectively. The maximum amplitudes of the sampled signal are 3.3 and 2.8 V respectively. According to Eq. 3, the transmission loss of the waveguide is $\alpha_{\mathrm{int}}$ = 0.71 dB/cm. The main waveguide loss includes scattering loss caused by side wall roughness, substrate leakage loss caused by silicon substrate with high refractive index, and light absorption loss caused by silicon dioxide. By optimizing the ICP etching procedure, such as changing the ICP power and introducing passivation gas, the roughness of the side wall can be reduced. By selecting SOI chips with thicker silicon dioxide layer, the leakage loss of the substrate can also be reduced. The absorption loss of silicon dioxide can be eliminated by suspending the silicon waveguide.

### 2.5 PCF of the silicon-on-insulator waveguide

For the 1 cm-long waveguide with N_2_ injected, the output can be expressed as

$$I_{\text{o}ut1}^{\prime }=I_{\text{in}}\exp\left(-\alpha_{\mathrm{int}}L_{1}\right)$$ (4)

Through Eqs. 1 and 4, the absorbance of CH_4_ can be expressed as

$$\text{Absorbance}=-\ln\frac{I_{\text{out}1}}{I_{\text{out}1}^{\prime }}=\Gamma \alpha cL_{1}$$ (5)

At a concentration of *c* = 10%, the direct absorption spectrum was measured and the absorbance of CH_4_ was obtained, which is 0.23314. Based on the high-resolution transmission (HITRAN) database, the absorption coefficient *α*_CH4_ = 10 cm^−1^. Then the actual PCF of the waveguide is determined to be 23.31%, which is lower than the simulation result of 34.3%. This can be attributed to the fabrication error of the SOI waveguide, leading to a changed mode filed distribution and effective refractive index.

## 3 Waveguide sensor performance using direct absorption spectroscopy

### 3.1 Calibration of CH_4_ sensor using direct absorption spectroscopy

**FIGURE 3:**
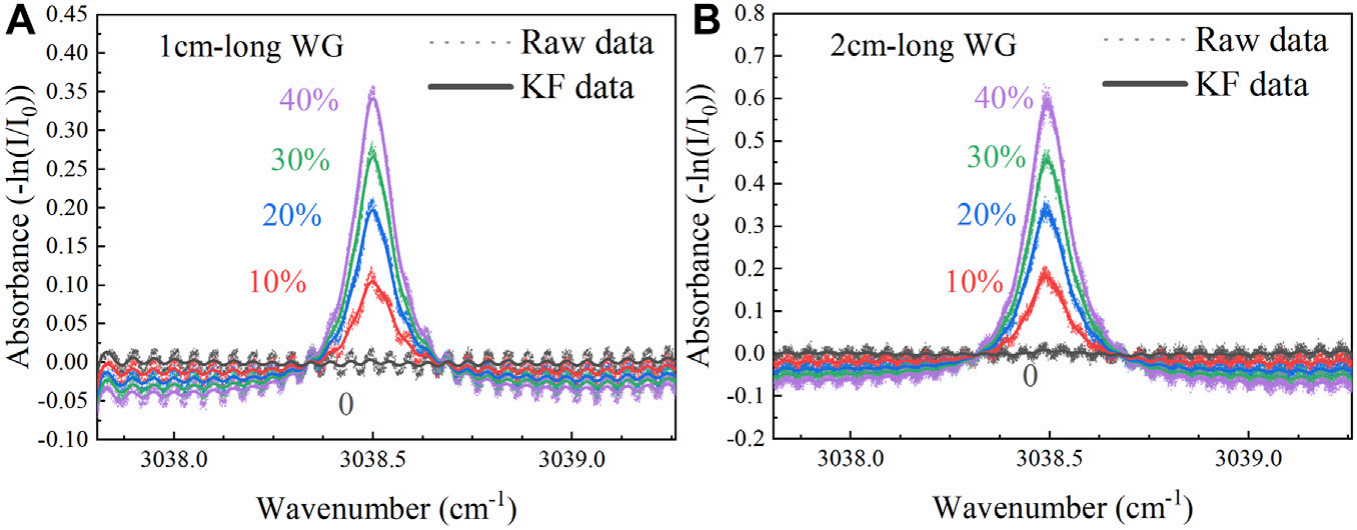
**(A)** Absorbance of the 1 cm-long waveguide under different CH_4_ concentration levels of 0, 10, 20, 30 and 40%. **(B)** Absorbance of the 2 cm-long waveguide under different CH_4_ concentration levels of 0, 10, 20, 30 and 40%.

In order to scan the absorption line of CH_4_ at 3,038.5 cm^−1^, the operating temperature of the ICL was set to 15°C. A triangular-wave scan signal with an initial voltage of 0.481 V and an amplitude of 0.095 V was applied to the ICL driver, so that the emission wavelength of the ICL can sweep from 3,037.637 to 3,039.151 cm^−1^. The sampling rate of the DAQ card was 5 kHz, resulting in 2 × 10^4^ data points in each scanning cycle, of which the 1 × 10^4^ data points corresponding to the falling slope signal in the second half of the cycle were used for DAS. Through a gas mixing system (Series 4,000, Environics), pure CH_4_ was diluted with nitrogen (N_2_) to form a gas mixture with CH_4_ concentration levels from 0 to 40%. At each concentration, the gas mixture was continuously injected into the gas cell for 2 min. At the sampling time of 0.2 s, the signal waveform and maximum absorbance were recorded, respectively. Figures 3A,B show the absorbance of the 1 cm-long waveguide and the 2 cm-long waveguide under different CH_4_ concentration levels (0, 10, 20, 30 and 40%), respectively.

**FIGURE 4:**
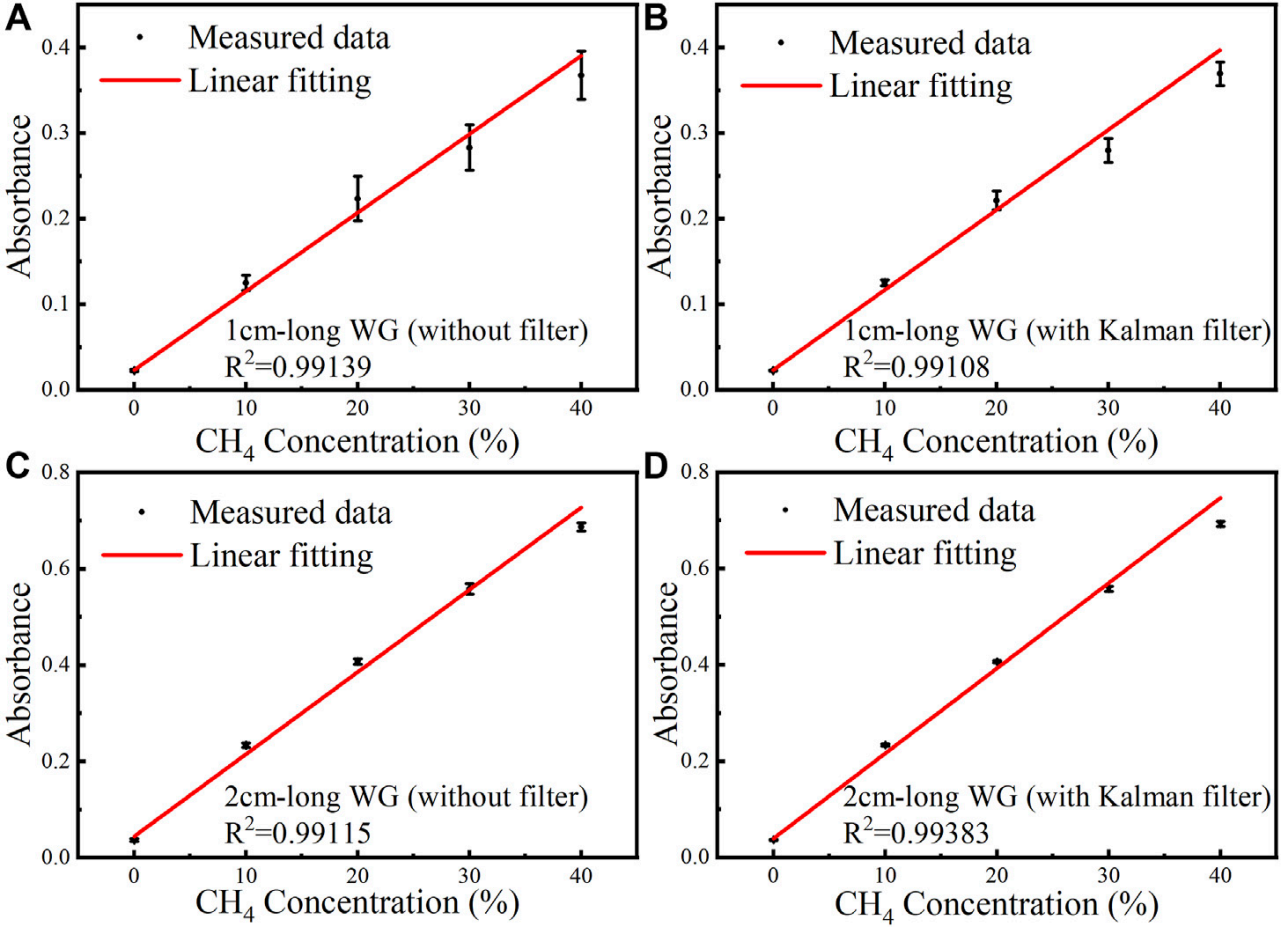
Under different CH_4_ concentration levels of 0, 10, 20, 30 and 40%, measured data and linear fitting curve of the absorbance versus CH_4_ concentration for the 1 cm-long waveguide **(A)** without Kalman filter and **(B)** with Kalman filter. Measured data and linear fitting curve of the absorbance versus CH_4_ concentration for the 2 cm-long waveguide **(C)** without Kalman filter and **(D)** with Kalman filter.

For the two waveguides with different lengths, under the conditions of using and without using Kalman filter, the average value of the absorbance at different concentration levels are shown in Figure 4A linear fitting was also carried out based on the experimental data, as shown in Figure 4. The *R* ^2^ of the linear fit is >99%. The error bars are different for different waveguide lengths and different CH_4_ concentration levels, due to different waveguide loss, optical output power and signal to noise ratio. Through Kalman filter, the signal fluctuation is suppressed and the error bars become smaller.

The linear fitting equations between the averaged absorbance and CH_4_ concentration of the 1 cm-long waveguide without and with Kalman filter are expressed by Eq. 6A and Eq. 6B. The linear fitting equations for the 2 cm-long waveguide sensor are expressed by Eq. 7A and Eq. 7B.

$${\text{Amp}}_{\text{DAS,}1\text{cm}}=9.18348\times 10^{-7}c\mathrm{+0}\mathrm{.02293}$$ (6A)

$${\text{Amp}}_{\text{DAS,}1\text{cm,Kalman}}=9.35487\times 10^{-7}c\mathrm{+0}\mathrm{.02283}$$ (6B)

$${\text{Amp}}_{\text{DAS,}2\text{cm}}=1.70741\times 10^{-6}c\mathrm{+0}\mathrm{.04371}$$ (7A)

$${\text{Amp}}_{\text{DAS}\text{.}2\text{cm,Kalman}}=1.76848\times 10^{-6}c\mathrm{+0}\mathrm{.03888}$$ (7B)

### 3.2 Stability

**FIGURE 5:**
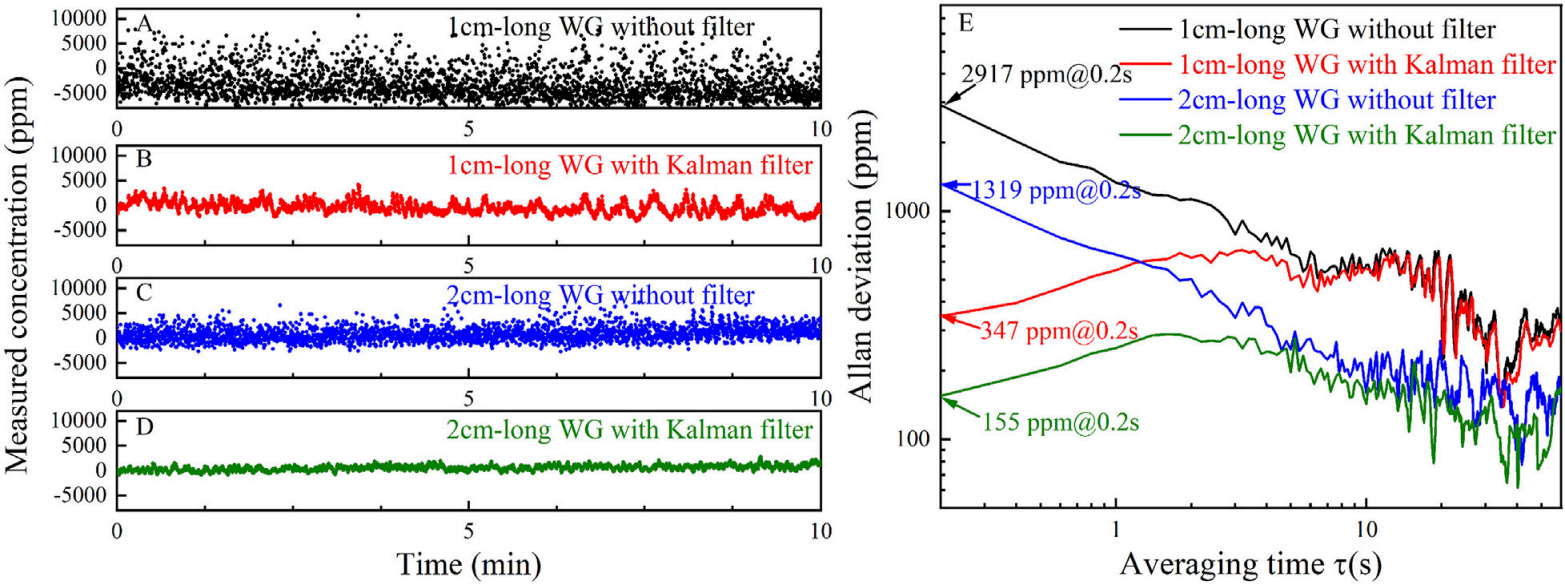
**(A)** Measured CH_4_ concentration levels of the 1 cm-long waveguide **(A)** without Kalman filter and **(B)** with Kalman filter by injecting pure N_2_ for 10 min. Measured CH_4_ concentration levels of the 2 cm-long waveguide **(C)** without Kalman filter and **(D)** with Kalman filter by injecting pure N_2_ for 10 min. **(E)** Allan deviation analysis based on the data shown in **(A–D)**.

In order to evaluate the stability and noise level of the system, pure N_2_ was injected into the gas cell. The absorbance was sampled for 15 min with a sampling period of 0.2 s. The curve of the calculated CH_4_ concentration levels versus sampling time is shown in Figures 5A–D. The Allan deviation of the sampled data was calculated, and its relation with the averaging time is shown in Figure 5E. Generally, with the increase of averaging time, the Gaussian white noise will be reduced, resulting in the reduction of the Allan deviation. However, due to the use of filtering in data sampling and processing, the effect of increasing the average time to reduce the Gaussian white noise is weakened. However, filtering cannot remove relatively low-frequency noise, it leads to the increase of Allan deviation in the initial stage with a small average time. Then the Allan deviation decreases because of the increased averaging time, which decreases the effect of the relatively low-frequency noise. As the averaging time increases to a certain extent, system drift begins to play a leading role, resulting in the increase of Allan deviation. Based on Allan deviation, the LoD of the 1 cm-long waveguide without and with Kalman filter are 2,917 ppm and 347 ppm, respectively, and those of the 2 cm-long waveguide are 1,319 ppm and 155 ppm, respectively, at an averaging time of 0.2 s.

## 4 Waveguide sensor performance using wavelength modulation spectroscopy

### 4.1 Modulation amplitude optimization

**FIGURE 6:**
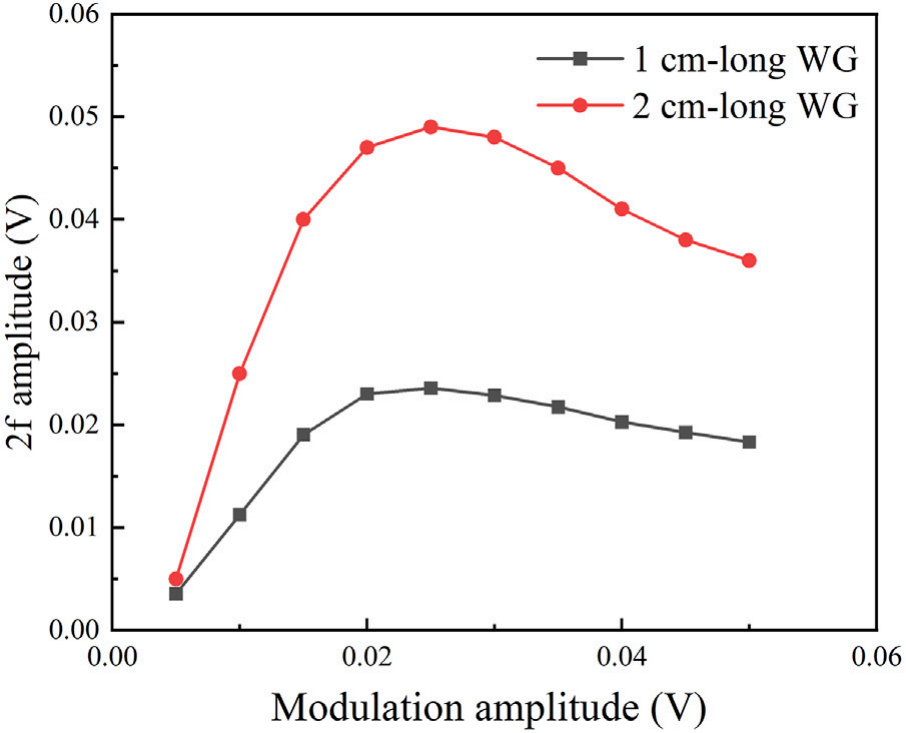
The measured 2f signal amplitude as a function of the modulation amplitude of the sinewave signal at a CH_4_ concentration level of 10^5^ ppmv for the 1 cm- and 2 cm-long waveguide.

In order to achieve a high signal-to-noise ratio (SNR), it is necessary to optimize the modulation amplitude. A mixture of CH_4_ and N_2_ was injected into the cell, where the CH_4_ concentration level is 10%. Under different modulation amplitudes, the amplitudes of 2f signal for the 1cm and 2 cm-long waveguides were recorded, as shown in Figure 6. For the two waveguides with different lengths, the maximum amplitude of the 2f signal was obtained when the modulation amplitude is 0.025 V. Because the amplitude of the 2f signal has a linear relationship with gas concentration, it is reasonable to optimize the modulation amplitude at one concentration level. Therefore, a modulation amplitude of 0.025 V was adopted in the WMS measurement.

### 4.2 Calibration of CH_4_ sensor using wavelength modulation spectroscopy

**FIGURE 7:**
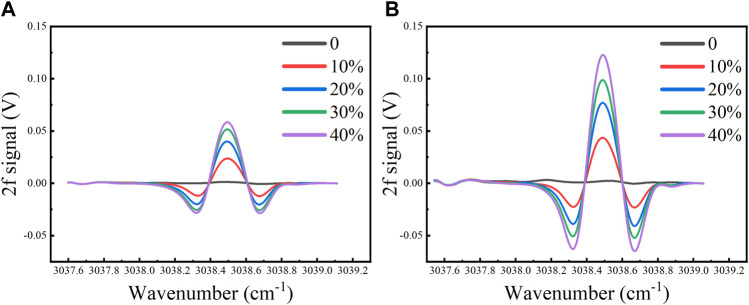
The measured 2f signal under CH_4_ concentration levels of 0, 10, 20, 30 and 40% for the **(A)** 1 cm-long and **(B)** 2 cm-long waveguide without Kalman filter.

In the first half cycle of the scan signal, there are 1 × 10^4^ data points for the rising ramp signal. A high-frequency sinusoidal modulation signal with a frequency of 5 kHz was superimposed on the triangular wave scan signal. At a sampling time interval of 0.2 s, the 2f signal waveform and its amplitude were recorded, respectively. Under different concentration levels (0, 10, 20, 30 and 40%), the 2f signal of the 1 cm and 2 cm-long waveguides without filter are shown in Figure 7.

**FIGURE 8:**
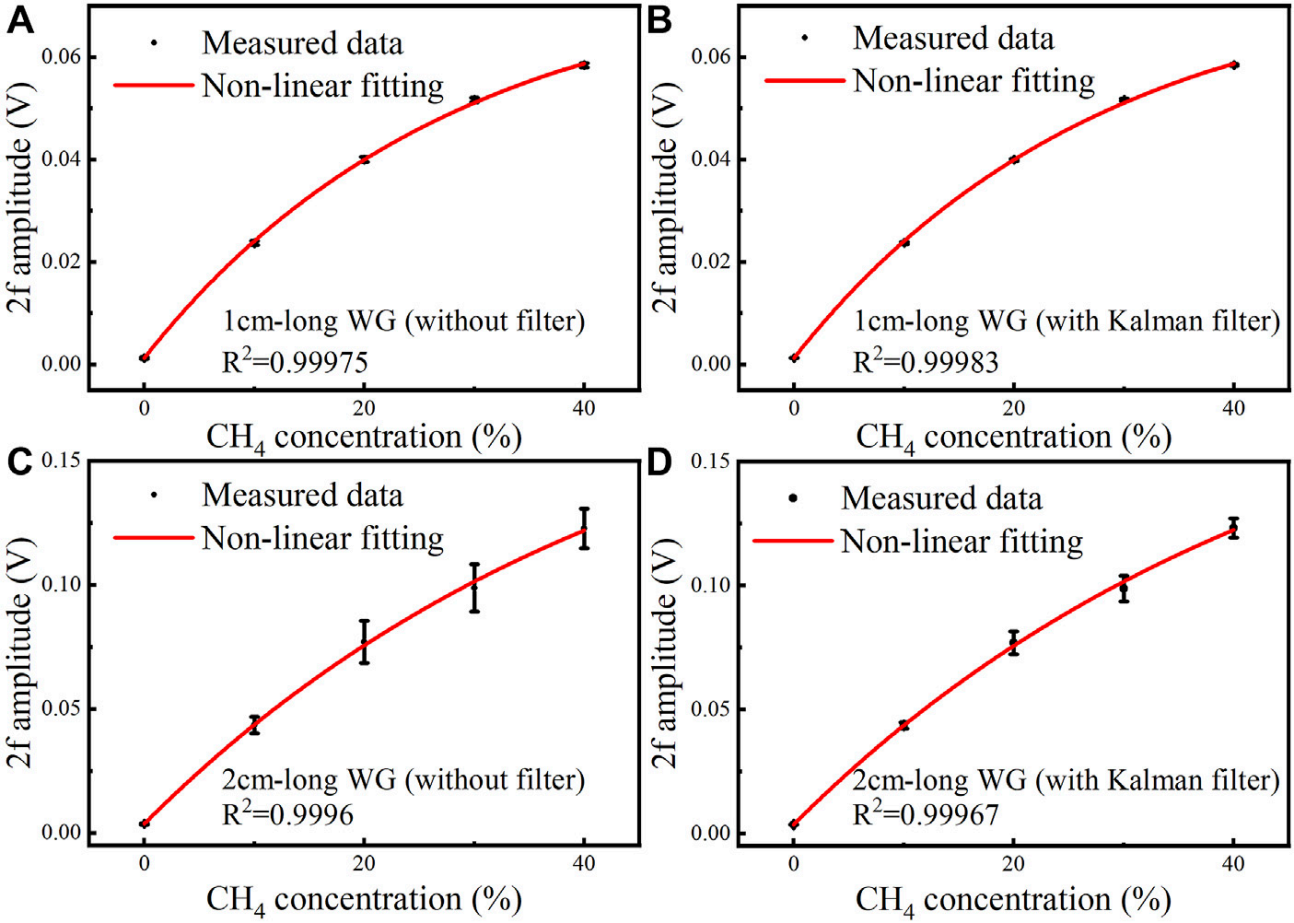
Measured data and non-linear fitting curve of the 2f signal amplitude versus CH_4_ concentration of the 1 cm-long waveguide under different CH_4_ concentration levels of 0, 10, 20, 30 and 40% **(A)** without Kalman filter and **(B)** with Kalman filter. Measured data and non-linear fitting curve of the 2f signal amplitude versus CH_4_ concentration of the 2 cm-long waveguide under different CH_4_ concentration levels of 0, 10, 20, 30 and 40% **(C)** without Kalman filter and **(D)** with Kalman filter.

At high CH_4_ concentration levels, the 2f signal amplitude shows a nonlinear trend. The 2f signal amplitude at different concentration levels were fitted non-linearly. The relationship between the 2f signal amplitude and CH_4_ concentration is shown in Figure 8, with *R* ^2^ > 0.999.

The non-linear fitting equations between the averaged 2f signal amplitude and CH_4_ concentration for the 1 cm-long waveguide without and with Kalman filter are expressed by Eq. 8A and Eq. 8B, respectively. The non-linear fitting equations of the 2 cm-long waveguide without and with Kalman filter are shown in Eq. 9A and Eq. 9B, respectively.

$${\text{Max}\left(2\text{f}\right)}_{1\text{cm}}=-0.07533\times \exp\left(-3.60497\times 10^{-6}c\right)+0.07658$$ (8A)

$${\text{Max}\left(2\text{f}\right)}_{1\text{cm,Kalman}}=-0.07528\times \exp\left(-3.60501\times 10^{-6}c\right)+0.07655$$ (8B)

$${\text{Max}\left(2\text{f}\right)}_{2\text{cm}}=-0.20228\times \exp\left(-2.19868\times 10^{-6}c\right)+0.20588$$ (9A)

$${\text{Max}\left(2\text{f}\right)}_{2\text{cm,Kalman}}=-0.20531\times \exp\left(-2.16122\times 10^{-6}c\right)+0.20891$$ (9B)

### 4.3 System stability

**FIGURE 9:**
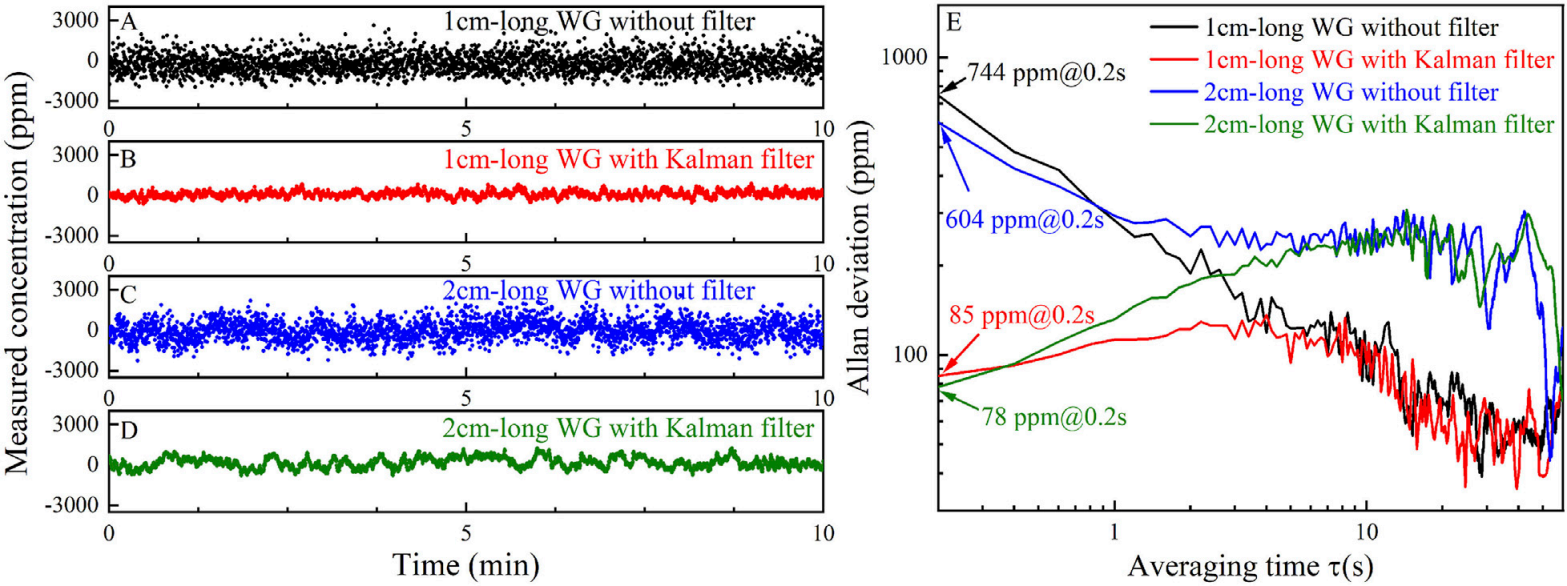
Measured CH_4_ concentration levels in N_2_ environment using the 1 cm-long waveguide **(A)** without Kalman filter and **(B)** with Kalman filter. Measured CH_4_ concentration levels in N_2_ environment using the 2 cm-long waveguide **(C)** without Kalman filter and **(D)** with Kalman filter. **(E)** Allan deviation analysis based on the data shown in **(A–D)**.

The stability measurement using WMS was carried out synchronously with DAS. The curve of the measured CH_4_ concentration levels versus sampling time is shown in Figures 9A–D. The Allan deviation based on the measured data was analyzed, and its relation with the averaging time is shown in Figure 9E. The LoD of the 1 cm-long waveguide without and with Kalman filter are 744 and 85 ppm, respectively, and those of the 2 cm-long waveguide without and with Kalman filter are 604 and 78 ppm, respectively, at an averaging time of 0.2 s. Therefore, by using WMS technique and Kalman filter, the LoD can be reduced from 347 ppm (using DAS) to 85 ppm for the 1 cm-long sensor, and the LoD can be reduced from 155 ppm (using DAS) to 78 ppm for the 2 cm-long sensor. This proves the denoising ability of the WMS technique.

### 4.4 Response time

**FIGURE 10:**
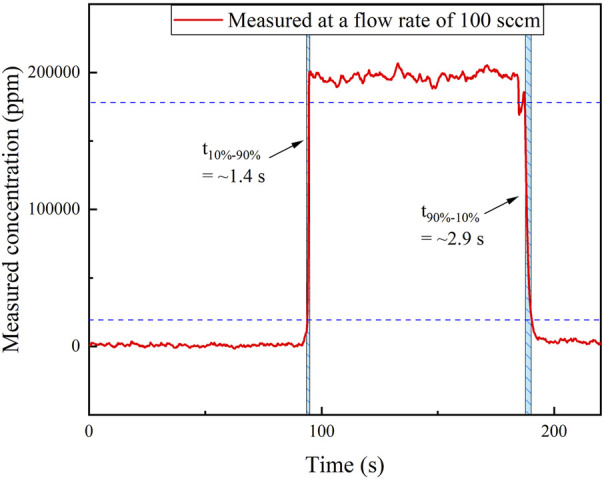
Response time measurement by switching the two gas samples with a concentration level of 0 ppm (N_2_) and 10%.

Experiment was performed to determine the 10–90% response time of the waveguide sensor. Pure N_2_ and 20% CH_4_ were repetitively injected into the gas cell, respectively, for a period of time. The two gas samples were exchanged twice, and the concentration level change was measured using the 1 cm-long waveguide. The results are shown in Figure 10. Under a gas flow rate of 100 sccm, the rise time from 10 to 90% of the target concentration is found to be ∼1.4 s, and the fall time from 90 to 10% is found to be ∼2.9 s.

## 5 Comparison and discussion

A performance comparison between the previously reported mid-infrared CH_4_ ChG optical waveguide sensor and the SOI optical waveguide CH_4_ sensor proposed in this paper is shown in Table 1. Compared with the reported ChG waveguide sensors (Han et al., 2016; Su et al., 2019), the prepared SOI waveguide sensor has lower optical loss (i.e., < 1 dB/cm). At the same time, due to the use of Kalman filter, the LoD measured by DAS and WMS are significantly reduced (as small as 100 ppm). When gas is injected into the PDMS gas cell, it will bring certain disturbance to the waveguide, resulting in additional noise. By adding Kalman filter, this kind of noise can be filtered out to a certain extent, and the signal-to-noise ratio (SNR) and LoD can be improved. In addition, because WMS has a certain suppressing effect on low-frequency noise, the LoD is lower than the DAS under the same waveguide length.

**TABLE 1:** Comparison among the reported CH_4_ waveguide sensors. KF: Kalman filter.

Refs	Wavelength (μm)	Technique	L (cm)	*α*_int_ (dB/cm)	PCF	Response time (s)	LoD (ppm)
ChG Han et al. (2016)	3.31	DAS	2	7	NA	NA	25,000 ^note2^
ChG Su et al. (2019)	3.31	DAS	0.5	8	NA	NA	10,000 ^note3^
SOI [this]	3.291	DAS, KF	1	0.71	23.31% (exp^note1^)	14, 10	347@0.2s
			2				155@0.2s
		WMS, KF	1				85@0.2s
			2				78@0.2s

**Note1**: this value was obtained through experiment. **Note2**: this value was obtained by monitoring the transmission change with different CH_4_ concentration levels at 3,310 nm, when the CH_4_ concentration decreased to 25,000 ppm, no attenuation of transport was observed compared to nitrogen atmosphere. **Note3**: this value was obtained by measuring the SNR., NA: not available.

In DAS and WMS measurements, we used two SOI waveguides with different lengths. Among them, the LoD for the 2 cm-long waveguide sensor is lower, because the 2 cm-long waveguide has a longer equivalent optical path under the same PCF. Due to the low transmission loss of SOI waveguide, it can be considered to fabricate longer waveguide to obtain lower LoD when the transmission loss allows.

## 6 Conclusion

Mid-infrared CH_4_ sensor system based on SOI waveguide was developed at 3,038.5 cm^−1^ for CH_4_ detection. SOI bend and straight waveguides as well as DAS and WMS measurement schemes were employed, and Kalman filter was used for noise suppression. CH_4_ sensing experiments were carried out using CH_4_ samples with five different concentration levels. The waveguide transmission loss was measured to be 0.71 dB/cm, and the PCF of the waveguide was experimentally determined to be 23.31%. Based on Allan deviation analysis on the 2 cm-long waveguide, by using DAS and WMS, the LoD are 155 and 78 ppm at an averaging time of 0.2 s, respectively, under the case of using Kalman filter. By further optimizing the PCF of the waveguide and appropriately increasing the length of the sensing waveguide, a lower detection limit is expected. The on-chip sensor shows potential applications in CH_4_ detection in chemical, oil and coal industries. In the future, by integrating the laser, detector and sensing waveguide on the same chip for more stable optical coupling and light transmission. Since no external light source and detector are required, the size of the sensing system will be significantly reduced and the portability will be improved.

## Data Availability

The original contributions presented in the study are included in the article/Supplementary Material, further inquiries can be directed to the corresponding authors.
